# Youth Physical Activity Patterns During School and Out-of-School Time

**DOI:** 10.3390/children5090118

**Published:** 2018-08-30

**Authors:** Pedro F. Saint-Maurice, Yang Bai, Spyridoula Vazou, Gregory Welk

**Affiliations:** 1 Department of Kinesiology, Iowa State University, Ames, IA 50011, USA; svazou@iastate.edu (S.V.); gwelk@iastate.edu (G.W.); 2 Department of Rehabilitation and Movement Science, University of Vermont, Burlington, VT 05403, USA; Yang.Bai@med.uvm.edu

**Keywords:** accelerometer, moderate-to-vigorous physical activity, season, Youth Physical Activity Measurement Study

## Abstract

This study describes age, sex, and season patterns in children’s physical activity behaviors during discrete time periods, both in school and at home. Participants were 135 elementary, 67 middle, and 89 high-school students (128 boys and 163 girls) involved in a larger school activity monitoring project. We examined time spent in moderate-to-vigorous physical activity (MVPA) at recess, physical education (PE), lunch, commuting to/from school, before-school, after-school, evening, and weekend segments. Differences in MVPA by age, sex, and season were examined using a three-way analysis of variance and separately for each individual segment. Moderate-to-vigorous physical activity levels varied by context and were higher during recess (15.4 ± 8.5 min) while at school, and on Saturdays (97.4 ± 89.5 min) when youth were out-of-school. Elementary children were more active than their older counterparts only during lunch time, after-school, and Sunday (*p* < 0.05). Boys were consistently more active than girls at all segments. Participants were only more active during non-winter than winter months during PE (*p* = 0.006), after-school (*p* < 0.001), and Sunday (*p* = 0.008) segments. These findings showed that activity levels in youth vary during the day and season. The segments reflect discrete time periods that can potentially be targeted and evaluated to promote physical activity in this population.

## 1. Introduction

The promotion of physical activity in youth remains a high priority among public health researchers with numerous agencies reinforcing the importance of concerted efforts to promote physical activity in this segment of the population [1,2]. Schools are frequently targeted as an ideal setting to reach and influence youth but it has proven difficult to disseminate evidence-based programs [3]. Recommendations have emphasized the advantages of integrated programming designed to impact different settings and periods throughout the day (e.g., physical education (PE), recess, lunch, commuting to and from school, after-school programs) [2,4].

To advance research on school activity promotion and integrated physical activity programming it is important to better understand how youth accumulate physical activity throughout the whole day and what settings are likely to have the greater margin for changes in physical activity levels. A few studies have demonstrated that physical activity at school typically accounts for the largest proportion of daily moderate-to-vigorous physical activity (MVPA) over the weekdays (i.e., ~25 min/day), followed by after-school programs (~15 min/day) [5]. Other research studies have described activity levels in more detail and across subgroups: during recess, lunch, and, to less extent, during PE classes and out-of-school periods, separately. Overall, these studies report that boys tend to be more active than girls and that activity accumulated during school (e.g., recess) and out-of-school periods (e.g., after-school, evening) tend to decrease as student’s transition from elementary to middle school grades [6,7,8,9,10,11]. However, a limitation of these past studies is that these did not capture activity levels during other important periods of the day, such as, before-school, or during commute to and from school. Additionally, it is also not known how seasonality can impact activity patterns during the day and at the various contexts where activity is likely to take place.

This study sought to fill this gap by quantitatively reporting how activity habits of school-aged youth are allocated across a full week. The data were collected as part of an ongoing project called the Youth Physical Activity Measurement Study (YPAMS) that uses a multimethod approach involving the collection of data with an online self-report tool as well as an established activity monitor [12,13]. The robust physical activity measurement protocol used in this study provides a useful way to examine the context of youth physical activity behavior. The study specifically examines age-, sex-, and season-related distributions of objectively measured physical activity accumulated throughout the week.

## 2. Materials and Methods

### 2.1. Participants

Our study used a convenience sample of schools from a Midwestern area in USA to participate in the YPAMS. We invited 29 schools and eight schools (two elementary, three middle, and three high schools) agreed to participate. A total of 625 students from 4th to 12th grade were informed about the study, received an enrollment package containing both consent and assent forms, and 343 participants agreed to participate (55% enrollment rate). This study was approved by the Institutional Review Board at Iowa State University (11–460 approved on 11/15/2011). All participants were required a signed consent form from parent or legal guardian and signed assent form in order to participate in the study.

### 2.2. Instruments 

#### SenseWear Armband

The SenseWear Armband (SWA) (BodyMedia, Inc., Pittsburgh, PA, USA) is a wireless pattern-recognition device that integrates motion sensor data with a variety of heat-related sensors, and demographic variables to estimate energy expenditure (EE) and minutes of physical activity [14]. This monitor was used to quantify physical activity estimates in our sample and has been shown to provide accurate estimates of physical activity and EE in this population [15,16,17]. The SWA was initialized with 1-min epochs and data were downloaded using InnerView v6.1 software (BodyMedia, Inc., Pittsburgh, PA, USA).

### 2.3. Design and Procedures

Data were collected as part of an ongoing cross-sectional study aimed at understanding youth activity patterns (http://www.youthactivitystudy.com). The specific data for the present study were obtained in a subsample of participants that completed replicate measurements. Each participant was assessed on two separate weeks (5–7 days apart) to obtain a more robust representation of their activity. Data collection was spread across a full academic year (Fall and Spring semester) and counterbalanced within each season among elementary, middle, and high-school participants, to enable variability and seasonality to be evaluated in all three age groups. At the first visit of each week of data collection, participants were provided with instructions on how to wear the SWA monitor and were given an activity log and asked to record activities during non-wear periods. The SWA monitors and respective logs were collected seven days later by the research team.

### 2.4. Data Processing

The data from the SWA monitors were downloaded from the InnerView software v6.1, exported in 1-min intervals, and processed using SAS v9.2 (SAS Institute, Cary, NC, USA). Accelerometer data were initially segmented into different windows of time to intentionally capture both school and out-of-school periods throughout the week. The distinction of school and out-of-school was particularly relevant considering the broad interest in promoting 60 min a day of physical activity among youth and that half of this amount should be accumulated at school while the remaining comes from out-of-school time [1]. School and out-of-school periods in our study were determined using the weekly schedule information of the participants obtained directly from schools and from pre-defined time blocks for weekdays after-school, evenings, Saturday, and Sunday periods. These periods were defined in accordance with our previous work [12]. More specifically, school segments included transportation to school, recess, PE, lunch, and transportation from school. Transportation to school was defined as starting 30 min before start time for school, while transportation from school was defined as going from the end of school time through 30 min after. recess, PE, and lunch were defined using schedule information provided by schools. Out-of-school segments included before-school, after-school, evening, Saturday, and Sunday. Before-school was defined as activity occurring during the weekdays and from 60 min before the start time for transportation to school until the start time for transportation to school. After-school schedule went from the end time for transportation from school through 6 p.m., while evening went from 6 p.m. through 10 p.m. Saturday and Sunday were each defined from 7 a.m. through 10 p.m. on respective weekend days.

Compliance was specific to each window of analyses (e.g., recess, PE, after-school) and included standard data reduction procedures (70% of total window time, at least three valid sessions per week with valid data, except for PE, Sunday and Saturday, when only one valid session was required). For example, for recess, participants’ SWA data were included in the analyses if the participant wore the monitor 70% of the recess time on at least three different recess sessions throughout the week. Non-wear time activities obtained from the logs were imputed in the raw data sets and were given a matching metabolic equivalent task (MET) value obtained from the compendium [18,19] using recommended procedures [20]. Approximately 2% of the total minutes of MVPA in our sample were imputed. Minute-by-minute predicted METs generated by the SWA were classified into minutes of activity and were categorized as MVPA if ≥4.0 METs.

### 2.5. Data Analyses

Replicate measures obtained in the two separate weeks were first aggregated to reflect a more robust/stable representation of usual week activity and then screened for outliers (defined as ±2.5 standard deviations away from the mean). Anthropometric measures included height, weight and computed body mass index (BMI) percentiles using Centers for Disease and Control and Prevention growth charts.

Preliminary analysis examined age by sex within-subjects’ variability in MVPA patterns across the different segments (see Appendix A). The main set of analysis examined between-subjects activity patterns (%MVPA per segment) by age group, sex, and season using three-way analyses of variance (ANOVA). Three-way ANOVAs (age group X sex X season) were conducted separately for each individual segment. For simplicity, only main effects associated with each factor are reported. Age group was coded as elementary, middle, and high-school grades. Data collected between September and November were coded as the non-winter season, while data collected between November and March was coded as the winter season. When relevant, minutes of MVPA were also provided for descriptive purposes in order to facilitate the interpretation of the distributions. Follow-up analyses for age group were performed using Tukey *t*-tests and the level of significance was set at an α of 0.01. All analyses were performed using SAS v9.2. (SAS Institute).

## 3. Results

From the 343 participants that agreed to participate, there were 291 participants (84.8% of the sample) that met the compliance criteria on at least one of the segmented activity windows considered in our study. Our analytical sample included: 135 elementary, 67 middle, and 89 high-school participants for the age group comparisons; 128 boys and 163 girls for the sex comparisons; and 147 non-winter and 144 winter observations for the comparisons by season. Average age was equal to 9.7 ± 1.0, 11.7 ± 0.8, and 15.7 ± 1.2 years for elementary, middle, and high-school participants, respectively. On average, participants wore the accelerometer for 8.8 h a day during a week day (528.4 min) and for 12.8 h during a weekend day (768.7 min). Detailed descriptives for this sample have been published elsewhere [13]. Compliance information is provided in Table 1.

### 3.1. Overall Activity Patterns

School-related opportunities for activity included commuting, recess, PE, and lunch. Recess was only available for elementary and middle school participants and was associated with the highest accumulated time spent in MVPA (65.0% of recess time was spent in MVPA; 16.5 ± 9.2 min). The second most active period was during PE (31.4%; 13.9 ± 11.1 min). The proportion of MVPA time spent on transportation to and from schools was 21.7% (5.2 ± 4.2 min) and 25.1% (6.3 ± 4.5 min) and 15.2% of the time during lunch (3.5 ± 3.1 min) was spent in MVPA. Out-of-school related opportunities for activity included before-school time, after-school period, evenings (all three periods during weekdays), and both on Saturday and Sunday. The proportion of time spent in MVPA was higher during after-school (17.8%; 22.5 ± 17.8 min) and lower at during the weekend (~11%; 87.7 min on Saturday and 86.8 min on Sunday). The proportion of MVPA were 11.8% and 13.2% during before-school and evening periods, respectively.

### 3.2. Activity Patterns by Age

**Figure 1:**
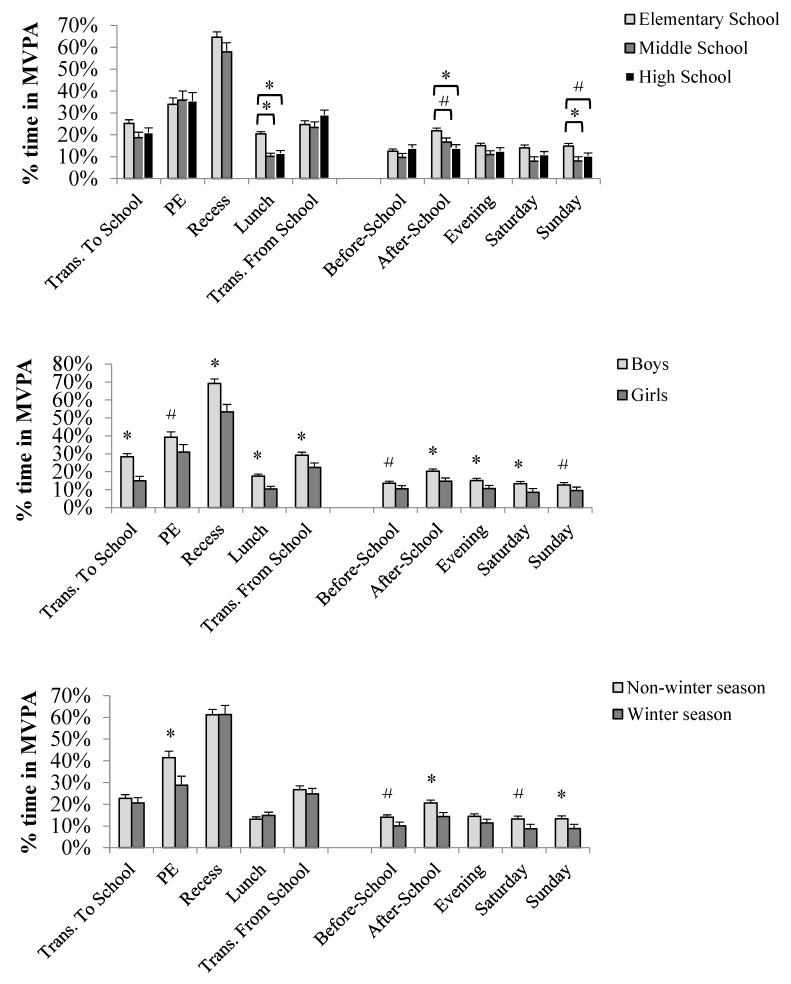
Week activity patterns by age group (**top**); sex (**middle**); and season (**bottom**). MVPA: moderate-to-vigorous physical activity. PE: physical education. * Indicates differences with *p* < 0.01. **#** Indicates differences with *p* < 0.05.

Age group was only significantly associated with activity accumulated at school during lunch time (*F* (2,258) = 20.06, *p* < 0.001). Activity during lunch was significantly higher among elementary school participants when compared with both middle (*t* (258) = 5.58, *p* < 0.001) and high-school (*t* (258) = 5.24, *p* < 0.001) children (Figure 1—top). Elementary children spent 20.4 ± 1.0% (5.0 ± 0.3 min) of their lunch time in MVPA while middle and high-school participants spent 10.1 ± 1.4% (2.8 ± 0.4 min), and 11.6 ± 1.3 (3.1 ± 0.3 min), respectively.

The same comparisons for activity out-of-school indicated that elementary children were more active than their older peers during after-school (*F* (2,244) = 7.25, *p* < 0.001), and also on Sunday (*F* (2,129) = 5.38, *p* = 0.006) (Figure 1—top). Elementary children were more active than their high-school peers (*t* (244) = 3.76, *p* < 0.001) and presented suggestive differences when compared with middle school participants (*t* (244) = 2.22, *p* = 0.03). Activity during after-school resulted in 21.8 ± 1.3% (27.8 ± 1.6 min), 16.7 ± 1.8% (21.9 ± 2.5 min), and 13.9 ± 1.6% (20.9 ± 2.2 min), for elementary, middle school, and high-school, respectively. Activity on Sunday was also higher for elementary school children when compared with middle school (*t* (129) = 3.00, *p* < 0.001; 14.8 ± 1.3% vs. 8.1 ± 1.9%) and approximated significance when compared with high-school participants (*t* (129) = 2.42, *p* < 0.017; 10.4 ± 1.3%). The distributions were equivalent to 106.4 ± 8.6, 74.5 ± 11.6, and 71.3 ± 9.5 min for elementary, middle, and high-school participants, respectively.

### 3.3. Activity Patterns by Sex

Boys took more advantage of school-related activity opportunities than girls. Percent time in MVPA was higher in boys for all school segments with the exception of PE (*F* (1,205) = 4.74, *p* = 0.031) (Figure 1—middle). Percent time in MVPA per PE class was equal to 39.3 ± 2.3% for boys, 31.0 ± 2.6% for girls, which is equivalent to 13.1 ± 1.1 and 11.3 ± 1.0 min of MVPA, for boys and girls, respectively. Percent time in recess was equal to 69.2 ± 0.0% and 53.3 ± 0.0% for boys and girls, respectively (boys: 13.4 ± 0.7; girls: 9.9 ± 0.7, minutes of MVPA per recess break).

Regarding out-of-school activities, boys were consistently more active than girls except before-school (*F* (1,185) = 4.54, *p* = 0.034) (6.2 ± 0.6 min for boys vs. 4.9 ± 0.5 min of MVPA for girls) and on Sunday (*F* (1,129) = 3.81, *p* = 0.053) (92.9 ± 8.6 for boys and 75.2 ± 6.9 for girls) (Figure 1—middle). Accumulated minutes of MVPA for boys resulted in 27.1 ± 1.7, and 32.3 ± 2.4 min during after-school and evening time, respectively, while girls accumulated 20.0 ± 1.5 and 24.3 ± 2.2 min of MVPA for the same time periods. The minutes of MVPA on Saturday for boys was equal to 100.1 ± 10.6 while girls accumulated 78.2 ± 9.0 min.

### 3.4. Activity Patterns by Season

Seasonal differences were significant at school-related opportunities during PE (*F* (1,205) = 7.85, *p* = 0.006) and at some out-of-school periods, namely, after-school (*F* (1,244) = 11.24, *p* < 0.001), and Sunday (*F* (1,129) = 7.24, *p* = 0.008) (Figure 1—bottom). Minutes of MVPA at PE during the non-winter season was approximately two times higher than PE activity accumulated during the winter season (15.7 ± 0.9 vs. 8.9 ± 1.3).

## 4. Discussion

Our study sought to characterize physical activity patterns among children and adolescents in discrete time periods including in school and out-of-school settings, weekdays and weekend, winter and non-winter periods. We found that overall, PE and recess provided the highest contribution for total minutes of daily activity during in-school time, while after-school and weekend had the greatest contributions for total during out-of-school time. Middle and high-school participants were less active than elementary students during lunch, after-school, and Sunday while boys were consistently more active than girls across all time segments. Activity levels at out-of-school settings were generally higher during non-winter months when compared to winter; however, there were no clear seasonality differences in MVPA for in-school settings.

We found age-related differences in MVPA during commute to school, lunch, after-school, evening, Saturday, and Sunday but surprisingly, there were no significant differences in MVPA during PE time. This can be partly explained by the structured nature of PE that is likely to engage students of different age groups in similar manner to participate in the planned activities. Unlike PE, there are several opportunities for unstructured physical activity spread throughout the school-day. These unstructured periods can provide greater room to implement physical activity promotion strategies, especially for older children and adolescents. In this study we showed that youth accumulated the lowest number of minutes in MVPA during lunch and commuting periods to/from school, while a greater portion of recess is usually spent in MVPA (~65%). This finding suggests that school-based activity interventions have potential to increase activity levels in settings such as commuting periods (e.g., active commuting) and during lunch if there is time available for children to be active. We also examined out-of-school opportunities as well as Saturday and Sunday physical activity patterns separately from weekdays. The activity pattern on Saturday and Sunday was relatively consistent in our study and resulted in the highest amount of activity accumulated in a day (~87 min/day). Although varied approaches have been taken to examine the weekend vs. weekdays physical activity patterns among youth, the consensus is that weekends are one of the particular periods that youth can benefit the most from appropriate intervention [7,21,22,23]. Also, during out-of-school time, we found that only 12–13% of the time in the evening and before-school was spent in MVPA. This finding also suggests that future interventions have potential to increase physical activity levels during these periods.

The seasonality of physical activity has been less frequently investigated in the literature but it is expected that weather is likely to influence youth physical activity. We found that children and adolescents were more active during the non-winter season, however, most of these differences were limited to out-of-school settings. Midwestern cities with cold winters are also more likely to be equipped with good indoor infrastructures that allow youth to play when the weather does not permit to be outside (i.e., under extreme weather conditions). Our findings are to some extent aligned with the previous literature. A systematic review of 16 studies examined the seasonal variations in children’s physical activity and found that the majority of studies (12 out of 16) reported seasonal differences and that these were moderated by other factors such as sex, ecology, geography or climate [24]. McCrorie et al. reported higher activity during summer season for Canadian adolescents, particularly between 5 p.m. to 9 p.m. after-school hours [25] while Silva et al. [26] found that children’s physical activity increased from winter to summer regardless of sex. Duncan et al. found that 10 °C increase in temperature was associated with over 1500 more steps per day in children [27]. The nature of our design did not allow a structured evaluation of the direct contribution of weather but it is clear that seasonality influences youth activity patterns.

The main contribution of the study is in providing descriptive information about youth physical activity behaviors across the most relevant in-school and out-of-school opportunities for youth to be active. The results demonstrate that activity levels vary across periods during the week (school vs. out-of-school) and suggest the need to better understand how these periods can be used to impact activity. However, it is important to consider some limitations with our relatively small, convenience sample of youth from schools in the Midwest of the US. This limitation is particularly important for our seasonality comparisons, so it is likely premature to generalize our conclusions to geographical areas with more mild climates. The cross-sectional design to examine differences in physical activity due to age and season is also a limitation but we distributed data collection among grades with a counterbalanced approach to avoid systematic bias or order effects. We also worked closely with the PE teachers to recruit a broad representative sample of youth in the classes to minimize selection bias.

In conclusion, our study adds unique value to the literature by describing activity levels across various periods of the day, using accurate information on youth schedules, and using a robust physical activity measurement protocol. Physical activity data in our study was collected using a well-established activity monitor (SWA) and replicate measures of activity to minimize random error in our objective measure of physical activity and further improve our estimates. This was particularly important considering that week one and week two of data collected were only moderately correlated (week day: *r* = 0.32; weekend day: *r* = 0.43). Future studies should explore the contexts we identified throughout the day (i.e., school and out-of-school periods) and determine the impact of context-specific interventions on youth activity levels.

## Figures and Tables

**Table 1:** Moderate-to-vigorous physical activity per day/session for school, out-of-school, weekend, and respective segmented windows of activity.

	*n*	MVPA (in minutes)	MVPA/Wear Time Duration (in %)	%PAGA (in %)	Wear Time Duration ^#^ (in minutes)
Total per school day	171	40.4 ± 18.9	30.3	76.8	133.2 ± 36.5
Total per out-of-school day	159	56.9 ± 33.5	14.4	94.8	394.7 ± 27.2
Total per weekend	109	175.0 ± 133.0	11.3	145.%	1542.0 ± 90.6
Total per week	62	302.0 ± 186.3	14.4	71.9	2092 ± 106.7
**School Day Segments**					
Transportation to school ^1^	226	5.2 ± 4.2	21.7	8.7	24.0 ± 2.5
Recess ^2^	162	16.5 ± 9.2	65.0	27.5	25.4 ± 10.0
PE ^3^	210	13.9 ± 11.1	31.4	23.2	44.3 ± 32.0
Lunch ^4^	263	3.5 ± 3.1	15.2	5.8	23.0 ± 5.3
Transp. from school ^5^	268	6.3 ± 4.5	25.1	10.5	25.1 ± 2.2
**Out-of-School Day Segments**					
Before-school ^6^	190	6.2 ± 5.4	11.8	10.3	52.7 ± 2.6
After-school ^7^	249	22.5 ± 17.8	17.8	37.5	126.1 ± 29.6
Evening ^8^	202	28.8 ± 23.5	13.2	48.0	217.6 ± 9.1
**Weekend Segments**					
Saturday ^9^	132	87.7 ± 76.3	11.4	141.2	767.2 ± 53.4
Sunday ^10^	134	86.8 ± 70.9	11.2	144.7	770.4 ± 55.6

MVPA: moderate-to-vigorous physical activity; PAGA%: percent equivalent to the physical activity guidelines of 60 min/day of moderate-to-vigorous physical activity (PAGA% for total weekend was computed assuming 120 min/weekend as the recommended, while PAGA% for total week was computed using 420 min/week as recommended; PE: physical education; ^#^ Indicates average number of minutes of valid use per day. ^1^ Transportation to school: 30 min before start time for school, ^2^ recess: schedule provided by school, ^3^ PE: schedule provided by school, ^4^ lunch: schedule provided by school, ^5^ transportation from school: from end time for school through 30 min after end time for school, ^6^ before-school: from 60 min before start time for transportation to school through start time for transportation to school, ^7^ after-school: from end time for transportation from school through 6 p.m., ^8^ evening: from 6 p.m. through 10 p.m. on weekdays, ^9^ Saturday: from 7 a.m. through 10 p.m., ^10^ Sunday: from 7 a.m. through 10 p.m.

## Appendix A. Age by gender within-subjects’ variability in MVPA.

These supplementary analyses examined within-subjects’ variation in physical activity across each of the individual day segments. Intraclass correlation coefficients (ICCs) were computed using the Shrout-Fleiss method (Shrout and Fleiss 1979) with segments (e.g., afterschool, PE, etc.) as fixed effects, subjects as random effects, and with average minutes of MVPA per segment as the independent outcome variable. These ICCs can be interpreted similarly as Cronbach α coefficients—with values ranging from zero to one, with a higher coefficient indicating higher degree of consistency among weekly segments and vice versa.

We found that activity among the different windows of the week were moderately correlated with an ICC of 0.59, 0.56, and 0.50 for elementary, middle and high-school groups, respectively. Age group by gender ICCs ranged from 0.49 to 0.51 in boys and 0.43–0.64 in girls, suggesting that activity was moderately consistent among the multiple segments of the week but that there is still considerable variability across the day in this population.
